# The Planning of Fever Hospitals

**Published:** 1895-03-23

**Authors:** 


### The Institutional Workshop.

### HOSPITAL ADMINISTRATION.

### THE PLANNING OF FEVER HOSPITALS.

At a meeting of the Royal Institute of British Architects held on February 25th, a lecture on fever hospitals was given by Mr. T. W. Aldwinckle. As the result of his experience he laid down certain axioms in regard to hospital construction too numerous for us to detail here, but certain points arose in the paper and in the discussion which followed it to which we think it well to draw attention. One of the most important of these was that "the several buildings of an infectious hospital should be completely isolated, and stand perfectly free, without communicating corridors or covered way of any kind." Foreign experience has long favoured this arrangement, and Mr. Aldwinckle was able to point to a few English hospitals in which the system had been applied. The authorities at the hospitals he mentioned as being without corridors or covered ways had all assured him that the staff experienced no ill-effects or inconvenience whatever from their absence. The complete isolation of the buildings was recognised and prepared for, and the nurses and others dressed accordingly. In the discussion Mr. Percival Gordon Smith, architect to the Local Government Board, said that he had many years ago strongly advocated the abolition of corridors between hospital blocks; they were not only costly and unnecessary, but undesirable. Both Dr. McCombie and Dr. Goodall, medical officers at hospitals, said that there were advantages in covered corridors, maintaining that officers, nurses, and servants in fever hospitals were human beings after all, and should be treated with consideration. Dr. Downes, of the Local Government Board, thought the great drawback to corridors between wards was not so much the risk of conveying infection as of blocking the movement of currents of fresh air. The danger of fire spreading from one pavilion to another, by means of the corridors, seems not to have been mentioned, obvious though it must be to anyone who has inspected the temporary structures at Tottenham or Tooting.

The question of the most desirable size of a fever hospital is one of very considerable importance, and the lecturer suggested that 300 beds was a desirable maximum. There is no doubt that in many ways there is an apparent economy in larger establishments. There are various departments which are as necessary for a small hospital as for a large one, and which need not be very much larger for the one than for the other. Nevertheless, Mr. Aldwinckle hesitates to recommend the aggregation of large numbers of sick upon one site. In this he was supported in the discussion. The fact is, it is not altogether a question of economy in construction. The human element comes into play in regard to management, and the real limit of the size of a hospital is that which allows the medical superintendent to exercise personal control in all departments, and the matron to influence personally all her nurses and her servants. Mr. Percival G. Smith was of opinion that a limit ought to be fixed as to the size of these institutions, for while the relative cost of a large hospital was less than that of a small one there were limits to the powers of supervision of a medical superintendent. He thought a fever hospital of 500 beds much too large, and even that number was being exceeded in London. Mr. G. T. Hall, however, regarded a hospital of 500 beds as the best from an administrative and economical standpoint, while Dr. Downes thought the maximum size of hospital which could be managed by one medical superintendent was one of 400 beds, and he hoped the Metropolitan Asylums Board would not go above this limit. Many other interesting topics were discussed which we have not space at present to enter upon.

